# Subthalamic Nucleus Electrical Stimulation Modulates Calcium Activity of Nigral Astrocytes

**DOI:** 10.1371/journal.pone.0041793

**Published:** 2012-07-27

**Authors:** Elodie Barat, Sylvie Boisseau, Céline Bouyssières, Florence Appaix, Marc Savasta, Mireille Albrieux

**Affiliations:** 1 Institut National de la Santé et de la Recherche Médicale, U 836, Grenoble Institut des Neurosciences, Equipe Dynamique et Physiopathologie des Ganglions de la Base, Grenoble F-38043, France; 2 Université Joseph Fourier, Grenoble F- 38042, France; 3 Centre Hospitalier Universitaire de Grenoble, BP217, Grenoble F-38043, France; The Mental Health Research Institute of Victoria, The University of Melbourne, Australia

## Abstract

**Background:**

The substantia nigra pars reticulata (SNr) is a major output nucleus of the basal ganglia, delivering inhibitory efferents to the relay nuclei of the thalamus. Pathological hyperactivity of SNr neurons is known to be responsible for some motor disorders *e.g.* in Parkinson's disease. One way to restore this pathological activity is to electrically stimulate one of the SNr input, the excitatory subthalamic nucleus (STN), which has emerged as an effective treatment for parkinsonian patients. The neuronal network and signal processing of the basal ganglia are well known but, paradoxically, the role of astrocytes in the regulation of SNr activity has never been studied.

**Principal Findings:**

In this work, we developed a rat brain slice model to study the influence of spontaneous and induced excitability of afferent nuclei on SNr astrocytes calcium activity. Astrocytes represent the main cellular population in the SNr and display spontaneous calcium activities in basal conditions. Half of this activity is autonomous (*i.e.* independent of synaptic activity) while the other half is dependent on spontaneous glutamate and GABA release, probably controlled by the pace-maker activity of the pallido-nigral and subthalamo-nigral loops. Modification of the activity of the loops by STN electrical stimulation disrupted this astrocytic calcium excitability through an increase of glutamate and GABA releases. Astrocytic AMPA, mGlu and GABA_A_ receptors were involved in this effect.

**Significance:**

Astrocytes are now viewed as active components of neural networks but their role depends on the brain structure concerned. In the SNr, evoked activity prevails and autonomous calcium activity is lower than in the cortex or hippocampus. Our data therefore reflect a specific role of SNr astrocytes in sensing the STN-GPe-SNr loops activity and suggest that SNr astrocytes could potentially feedback on SNr neuronal activity. These findings have major implications given the position of SNr in the basal ganglia network.

## Introduction

The pars reticulata of the substantia nigra (SNr) is the main output nucleus of the basal ganglia network and conveys the final output signal to the thalamus and brain stem [1]. The GABAergic neurons of the SNr are tonically active and display spontaneous activity (around 10 Hz) that is partially independent of synaptic inputs [2]. Thus, the brain areas receiving inputs from the basal ganglia are, by default, under strong tonic inhibition. Brief changes in firing, often associated with movement, result in the disinhibition or further inhibition of the target nuclei neurons [3]. The SNr may therefore be considered as a gateway in the transmission of information to the motor and cognitive systems. This output nucleus mostly integrates inputs from the striatum, the external segment of globus pallidus (GPe) and the subthalamic nucleus (STN). Among its inputs, the excitatory STN and the inhibitory GPe play a particularly important role in the regulation of SNr activity as they form a feedback system engaged in synchronized bursting and constituting a basal ganglia pacemaker [4]. The STN occupies a crucial position in the functional architecture of the basal ganglia network since it is the only glutamatergic nucleus [5], [6]. Abnormal hyperactivity of the STN, consecutive to the loss of dopaminergic cells in the substantia nigra pars compacta, is thought to play a critical role in the expression of Parkinson's disease symptoms [7], [8]. High-frequency stimulation of the STN (STN-HFS) is currently the main surgical therapy for treatment of advanced Parkinson's disease. The cellular mechanisms of STN-HFS are still debated but it has been proposed that HFS could inactivate STN neurons [9], [10] or change their abnormal firing mode into recurrent bursting activity [11]. This normalization could reduce the hyperactivity of basal ganglia output structures, such as SNr [12], [13], [14]. STN-HFS may produce these effects by activating the axons of STN neurons, STN afferents or fibers passing close to the stimulation site, leading to both glutamate and GABA release within SNr [15], [16], [17]. As the final stage of information processing within the basal ganglia occurs mainly in the SNr, studying the regulation of the activity of this nucleus in physiopathological conditions is of major importance [18]. SNr properties for integrating neuronal network and signal processing of the basal ganglia are relatively well known but, paradoxically, the role of astrocytes in the regulation of SNr activity has never been studied.

Over the past decade, the dogma that astrocytes play a subsidiary role in the nervous system has become obsolete. Astrocytes are now viewed as active components of neural networks, as they are endowed with a great variety of voltage and ligand-operated ion channels [19]. An increasing body of evidence obtained the last few years has established the concept of the tripartite synapse, in which astrocytes play an active role by exchanging information with the synaptic elements [20], [21] concomitantly with classical homeostatic roles. This concept is based on the demonstration that astrocytes display a form of excitability based on intracellular calcium variations [22]. Neurotransmitters, such as glutamate and GABA, activate receptors on astrocytes [20], [23], [24], [25] generating calcium transients that propagate within the astrocyte population. In particular conditions [26], this increase in astrocytic calcium levels can induce the release of neuroactive substances, such as glutamate, ATP [20], [23] or D-serine [27], [28]. These gliotransmitters may modulate neuronal excitability [29], [30], [31] and the strength of synaptic transmission [20], [23], [32]. The general importance of bidirectional communication between astrocytes and neurons throughout the brain prompted us to study its specific contribution to SNr neuronal activity.

In order to address the role of astrocytes in the regulation of the SNr neuronal activity, we characterized SNr astrocyte excitability and its modulation by the GABA/glutamate balance regulated by the pallido-nigral and subthalamo-nigral loops. We developed a sagittal rat brain slice that (i) conserves the STN-GPe-SNr functional loops, (ii) allows physiological modulation of the activity of the loops and of GABA/glutamate balance within the SNr by stimulating the afferent nuclei. We found that SNr astrocytes displayed spontaneous calcium activity in basal conditions. This calcium activity was partly autonomous and partly dependent on the spontaneous release of GABA and glutamate. Interestingly, disturbing the glutamate/GABA balance by STN-HFS affected the SNr astrocytic calcium activity. These results suggest a specific interaction between neurons and astrocytes within SNr, modulated by the balance between excitation and inhibition.

## Results

### Preservation of STN-GPe-SNr pathways in rat sagittal brain slices

**Figure 1:**
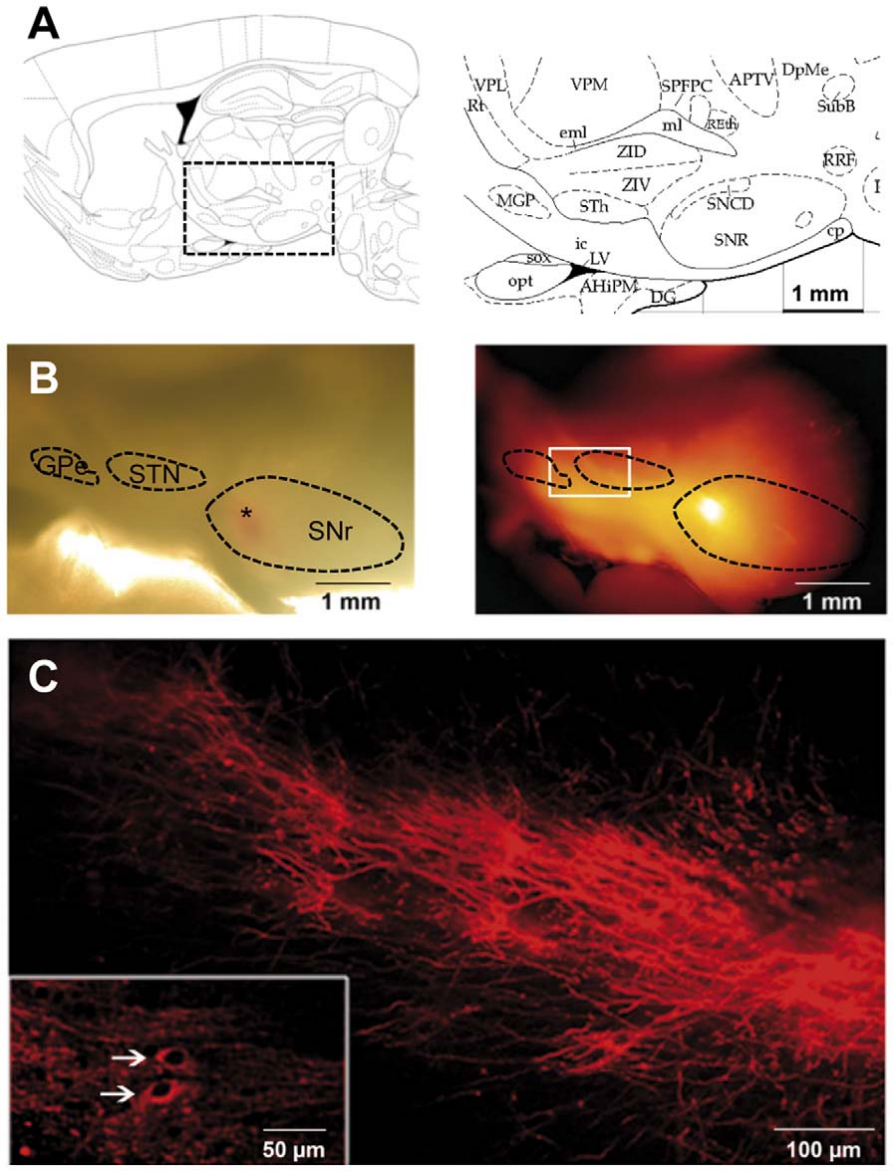
Subthalamo-nigral and pallido-nigral anatomic connections are preserved in sagittal slices. (**A**) Schematic diagram of the area of interest (right panel) in a sagittal brain slice (left panel, modified from [33]) showing the location of the STN (indicated as STh), GPe (MGP) and SNr (SNR). (**B**) A DiI crystal was placed in the SNr (depicted by the asterisk in the bright field image, left part) and the slice was incubated for 4 weeks at room temperature. DiI diffused towards STN and GPe (epifluorescence image on the right). (**C**) Higher magnification of the region indicated by the white rectangle on image B showing subthalamo-nigral and pallido-nigral projections. In some cases, DiI tracing showed retrograde labeling of some cells in the STN (inset, white arrows).

The pallido-nigral and subthalamo-nigral loops have a crucial influence on the spontaneous activity of SNr neurons and we wondered about their impact on astrocyte activity. The adult rat brain atlas suggested that these nuclei were present on the same sagittal plane, from 2.4 to 2.9 mm laterally to the midline, in adult rat (Fig. 1A) [33]. We performed such sagittal brain slices and introduced lipophilic tracers (DiI crystals) into the SNr or the STN to confirm that the subthalamo-nigral and pallido-nigral pathways were indeed preserved. The dye diffused for four weeks, resulting in retrograde and anterograde labeling of the persistent subthalamo-pallido-nigral pathways. When a crystal was placed in the SNr (Fig. 1B), bundles of labeled fibers could be seen coursing from the SNr towards the STN and probably also towards the GPe beacuse some of these fibers forked close to or within the STN [34] (Fig. 1C). We also detected some labeled soma within the STN (Fig. 1C, inset) meaning that at least some projecting STN neurons were intact. When a crystal was placed in the STN, two pathways running in opposite direction were observed, one in the rostrocaudal direction and the other one (usually more intense) in the caudorostral direction, suggesting connections towards SNr and GPe respectively (not shown). These observations confirmed that the glutamatergic subthalamo-nigral pathway and the GABAergic pallido-nigral pathway were, at least partly, preserved in these slices.

On acute slices, incubation with propidium iodide and Hoechst 33342 showed that 80±5% of the SNr cells were still alive 5 hours after slicing (*n* = 16 slices). Electrophysiological experiments were carried out to record the typical pace-maker activity of nigral GABAergic and dopaminergic neurons [2] in these sagittal slices (*n* = 15 neurons in a total of 7 slices). Patch-clamp recordings performed in sagittal plane by other groups had already shown that SNr neurons were affected by a complex pattern of excitatory glutamatergic and inhibitory GABAergic synaptic currents [34], [35]. Thus, in these conditions, GABAergic pallidal activity, glutamatergic STN activity and/or GABAergic intranigral activity may potentially contribute to the basal calcium activity of astrocytes in the SNr.

### Astrocytes constitute the main cell population in the SNr

**Figure 2:**
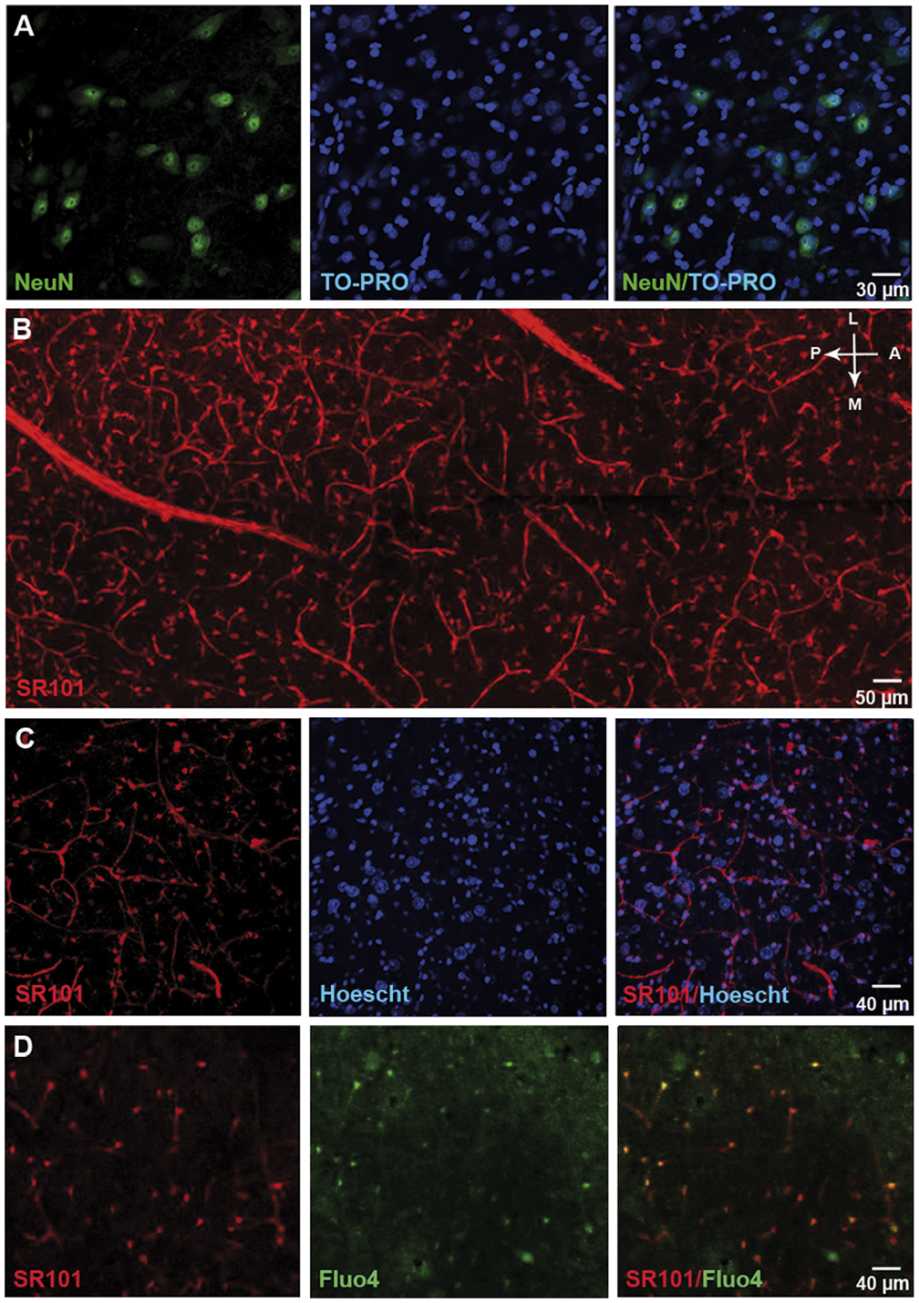
Characterization of SNr cell populations and Fluo-4 loaded cells. (**A**) NeuN staining (green) labels few cells within SNr. Nuclei are identified by TO-PRO staining (blue). Merged image showing the proportion of neurons within the SNr (right panel). (**B**) Two-photon multistack (40 slices at 2 µm spacing) mosaic reconstruction of SNr astrocytes and vessels in an acute brain slice 2 h after sulforhodamine 101 (10 mg/ml) intravenous injection (100 µl/50 g body weight). Antero-posterior (AP) and medio-lateral (ML) orientations are shown in the upper right part of the figure. (**C**) Bi-photon imaging of sulforhodamine 101 (red) and Hoescht 33342 (blue) in the SNr. Merged image showing the proportion of astrocytes within the SNr (right panel). (**D**) Representative confocal images of SR101-labeled (red) and Fluo-4-loaded (green) cells in the SNr of a sagittal acute slice of rat brain. Merged image showing sulforhodamine 101 and Fluo-4 staining within the SNr, confirming that most of the loaded cells are astrocytes.

The SNr contains two types of neurons: GABAergic and dopaminergic neurons [2]. GABAergic neurons predominate (about 90% of SNr neurons, [36]), forming the major output from the basal ganglia [37]. With an average of 1795±358 total cells/mm^2^ (*n* = 7 slices from 3 rats, estimated by nuclei staining with TO-PRO 3) throughout the whole SNr, immunohistochemical stereological analysis showed that neurons (as revealed by NeuN immunostaining) were heterogeneously distributed within the SNr and accounted for 12±3% of total SNr cells (Fig. 2A; *n* = 16 tissue sections from 4 rats). When we quantified the astrocyte population with S100B immunostaining, we found 42±3% of S100B-positive cells in the SNr (Fig. S1A; *n* = 6 tissue sections from 2 rats). As described in other brain structures [38], [39], however, we observed that S100B or GFAP are not co-expressed in all astrocytes within the SNr (*e.g.* 15±5% of S100B-positive cells were GFAP-positive and 51±3% of GFAP-positive cells were S100B-positive, *n* = 3 slices; Fig. S1B). Thus, this cell population might be underestimated on the basis of immunostainings. We therefore used an alternative strategy to precisely and specifically determine the proportion of astrocytes in the SNr. Intravital sulforhodamine dyes are known to be specifically targeted to the astrocyte population when injected intravenously in rats [40], [41]. Sulforhodamines cross the blood brain barrier and astrocytes are stained directly via their endfeet. We then achieved sagittal living brain slices and loaded them with intravital Hoechst 33342. Sulforhodamine 101 (SR101) staining showed astrocytes to be uniformly distributed throughout the SNr (Fig. 2B) with similar morphology when compared to S100B immunostaining (Fig. S1A). We found that SR101-stained astrocytes accounted for 65.0±7.2% of total SNr cells (*n* = 6 slices from 3 rats; 48 fields; Fig. 2C).

A major advantage of SR101 staining is that it can be combined with Fluo-4 AM loading in acute living slices for calcium imaging. Thus, we showed that among these SR101-stained astrocytes, 76.2±6.6% were loaded with Fluo-4 (*n* = 6 slices from 2 rats; Fig. 2D). Moreover, in our conditions, 98.0±2.2% small (less than 10 µm) Fluo-4 loaded cells were astrocytes (SR101 stained cells; *n* = 6 slices from 2 rats; Fig. 2D), indicating that almost all the cells subsequently recorded were astrocytes. Microscopic analysis of the Fluo-4 labeled slices confirmed that, compared to neurons, the astrocyte cell bodies were smaller and more strongly labeled by Fluo-4, probably due to their greater ability to take up or hydrolyze AM-ester dyes. This allowed us to distinguish between the two cell populations on morphological criteria, as already reported for mouse cortex [42].

The remaining SNr cells corresponded to other glial cell populations and were composed of 10±3% NG2 positive cells (*n* = 5 tissue sections from 2 rats), 13±4% oligodendroglial-derived lineage cells (Olig 2 immunostainings; *n* = 10 tissue sections from 3 rats) and 7±4% microglial cells (Ox-42 immunostainings; *n* = 6 tissue sections from 3 rats).

### Astrocytes display spontaneous calcium activities in physiological conditions

**Figure 3:**
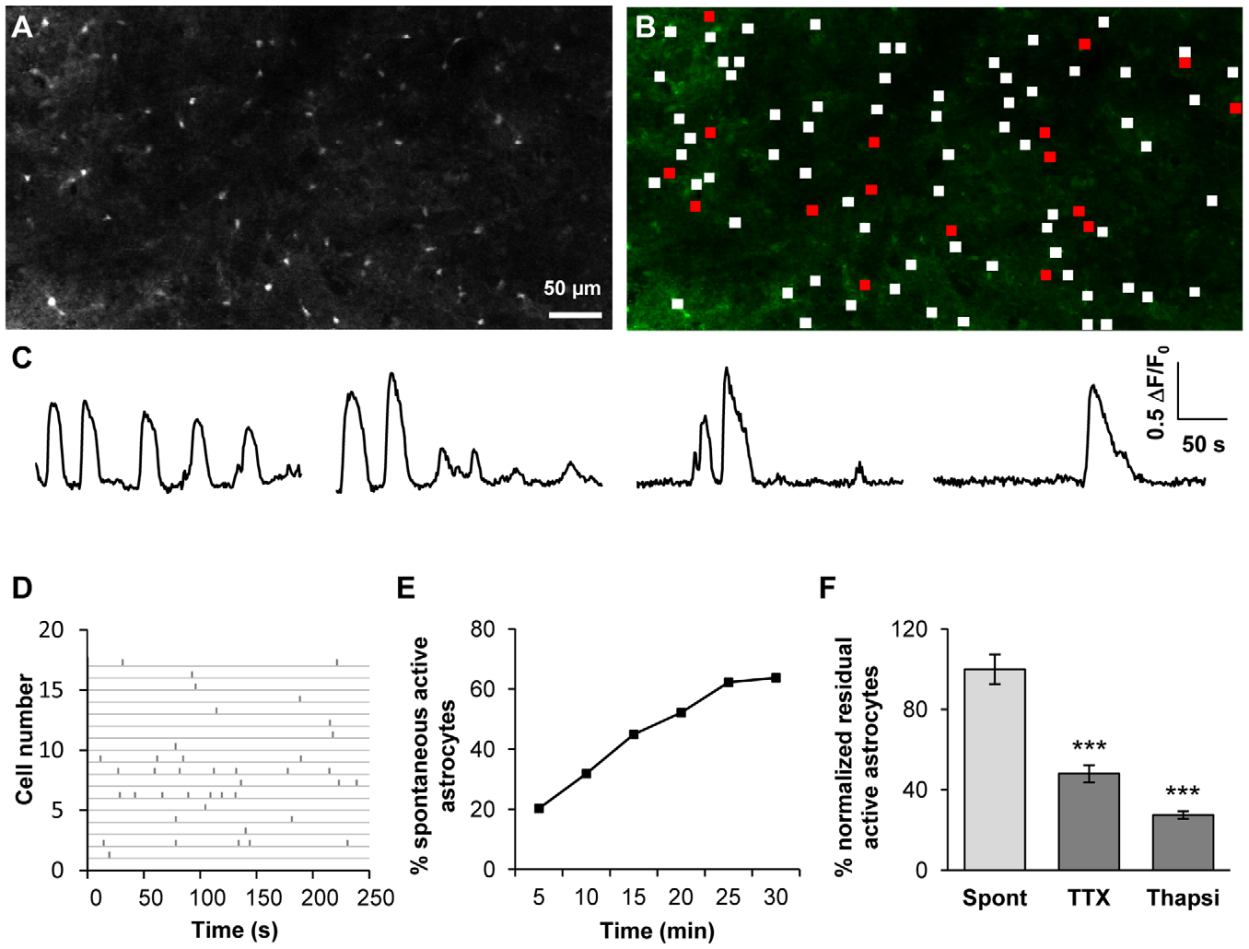
SNr astrocytes display spontaneous calcium activity that is partly dependent on neuronal activity. (**A**) Fluo-4 loaded cells in the SNr area of a rat sagittal acute slice. Only small cells (astrocytes, less than 10 µm in diameter) were loaded. GABAergic and dopaminergic neurons (with cell bodies from 20 to 40 µm in diameter [70]) were not labelled in most cases. (**B**) Distribution of active (red frames) and non active (white frames) ROIs within the slice shown in A. (**C**) Example of typical fluorescence variations recorded in four SNr astrocytes. (**D**) Raster plot of fluorescence peaks detected in active ROIs described in B. (**E**) Example of the cumulative progress of the proportion of active astrocytes during recording over 5, 10, 15, 20, 25 and 30 minutes. (**F**) Effect of 500 nM TTX (*n* = 13 slices; *p*<0.001) or 2 µM thapsigargin (Thapsi, *n* = 7 slices; *p*<0.001) on the spontaneous calcium activity of astrocytes in the SNr.

In order to assess the impact of astrocytes on SNr output activity, we first recorded spontaneous calcium activity using calcium imaging on rat sagittal brain slices at the subthalamo-pallido-nigral level. On average, individual movies (field of view around 600×300 µm) included 66.3±15.3 astrocytes (*n* = 54 slices; neurons were excluded on the basis of morphological criteria). In basal conditions, 30.8±14.5% of the SNr dye-loaded astrocytes displayed spontaneous calcium activity during a five-minute recording period (Fig. 3A–D; *n* = 54 slices from 29 animals, 3330 cells). This proportion increased with the recording period (64% astrocytes displayed calcium activity when observed for a 30 minute-period, Fig. 3E) suggesting that most of astrocytes display spontaneous activity. In active astrocytes, the calcium signal had a mean duration of 23.5±6.8 s with a mean amplitude of 0.19±0.06 (ΔF/F_0_, arbitrary units). It occurred at a frequency of 0.37±0.07 event/min (*n* = 54 slices, 3330 cells). Synchronous activity was observed in 2.6% spontaneous active astrocytes.

We assessed the autonomous part of spontaneous calcium activity recorded in astrocytes (*i.e.* independent of neuronal activity) by incubating slices with tetrodotoxin (TTX, 500 nM) to block neuronal transmission. The effect of TTX was analyzed on a 5 minute recording and compared to a previous recording of calcium activity performed in the same area just before treatment. The inhibitory effect was then compared to a control experiment, performed in the same conditions but without drugs, and normalized. In presence of TTX, the proportion of astrocytes displaying spontaneous activity was significantly lower (52.0±9.9% inhibition; *p*<0.001; *n* = 13 slices from 10 rats, 908 cells; Fig. 3F) indicating that astrocytic calcium activity partially depends on neuronal activity. In the residual active astrocytes, TTX had no effect on calcium activity parameters (such as frequency, duration and amplitude) suggesting that this remaining calcium activity is totally independent of neuronal activity. Moreover, a separate analysis of TTX-sensitive and TTX-insensitive cells performed in the first recording movie (*i.e.* without TTX) revealed no obvious difference in the pattern of calcium signaling between these two sub-populations. As in basal conditions, no synchrony between active cells was observed in the presence of TTX.

Incubation of slices with thapsigargin (2 µM during 30 minutes; a Ca^2+^ store depleting agent) significantly decreased the number of active astrocytes (72.4±9.1% inhibition; *p*<0.001; *n* = 7 slices from 5 rats; 738 cells; Fig. 3F). This suggests that Ca^2+^ stores constitute the main route for the astrocytic calcium activity but extracellular Ca^2+^ source may also be involved.

These data provide the first evidence of spontaneous calcium activity in the astrocytes of the SNr in basal conditions. Half of this spontaneous activity is autonomous; the other half depends on neuronal activity.

### Glutamate and GABA receptors mediate calcium signals in SNr astrocytes

**Figure 4:**
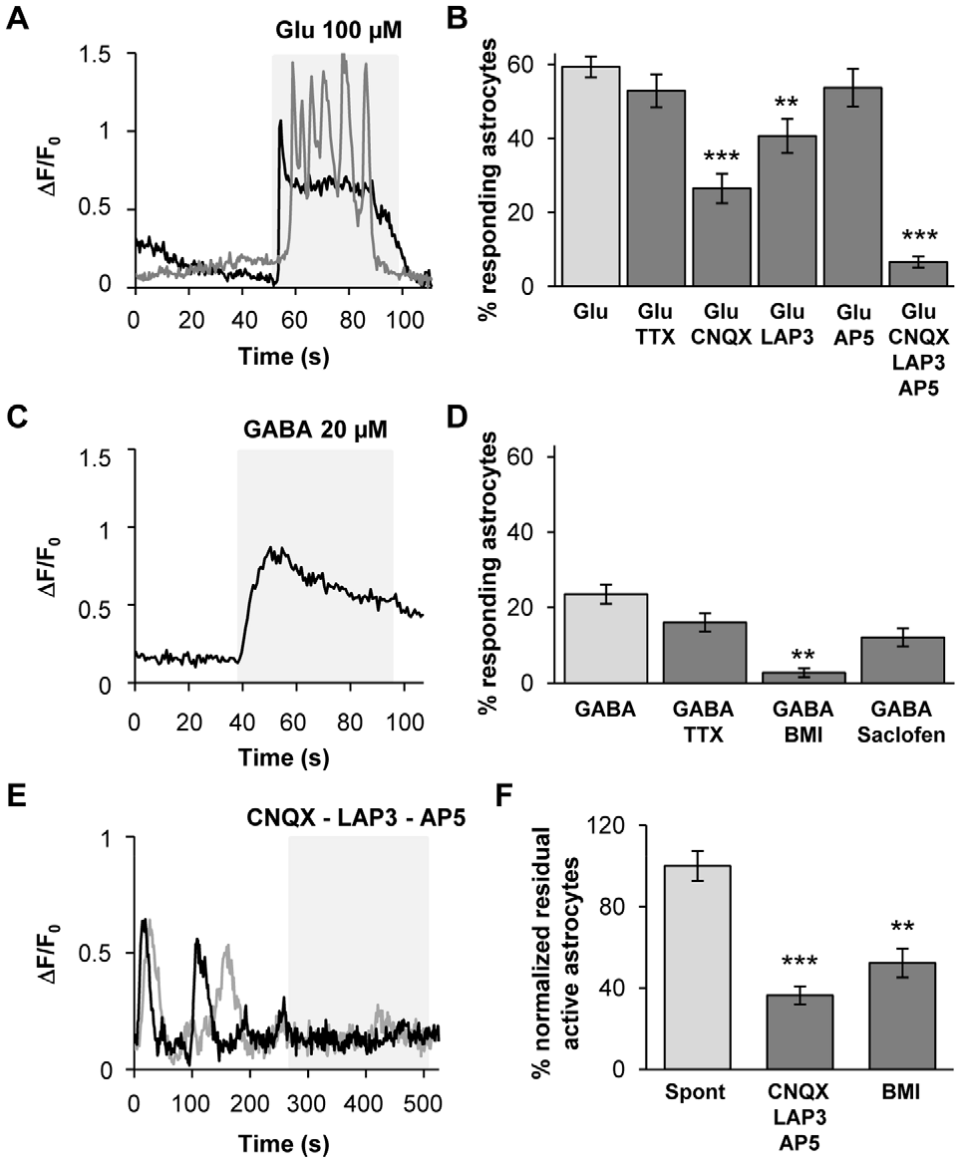
SNr astrocytes AMPA, mGlu and GABA_A_ receptors are involved in calcium spontaneous activity. (**A**) Example of two typical fluorescence variation profiles recorded in SNr astrocytes when 100 µM glutamate was perfused in the bath. (**B**) Histogram of the percentage of loaded SNr astrocytes responding to the application of 100 µM glutamate (*n* = 15) and the effect of 500 nM TTX (*n* = 12; *p* = 0.317), 10 µM CNQX (*n* = 8; *p*<0.001), 100 µM LAP-3 (*n* = 11; *p* = 0.005), 50 µM AP-5 (*n* = 4; *p* = 0.453) or a cocktail containing 10 µM CNQX+100 µM LAP-3+50 µM AP-5 (*n* = 4; *p* = 0.001) on this glutamate-induced effect. (**C**) Typical fluorescence variations recorded in a SNr astrocyte when 20 µM GABA was perfused in the bath. (**D**) Histogram of the percentage of loaded SNr astrocytes responding to the application of 20 µM GABA (*n* = 18) and the effect of 500 nM TTX (*n* = 7; *p* = 0.215), 20 µM BMI (*n* = 7; *p* = 0.02) or 100 µM saclofen (*n* = 8; *p* = 0.948) on this GABA-induced effect. (**E**) Typical fluorescence variations recorded in two SNr astrocytes before and after incubation with a cocktail containing 10 µM CNQX+100 µM LAP-3+50 µM AP-5. (**F**) Histogram showing the effect of a cocktail containing 10 µM CNQX+100 µM LAP-3+50 µM AP-5 (*n* = 10; *p*<0.001) or 20 µM BMI (*n* = 7; *p* = 0.003) on the spontaneous calcium activity of astrocytes in the SNr. The inhibitory effect is normalized with respect to control residual activity. *, *p*<0.05; **, *p*<0.01 and ***, *p*<0.001.

The most widely accepted mechanism for astrocytic Ca^2+^ increase is dependent on G protein-coupled metabotropic receptors that leads to release of intracellular InsP_3_-activated Ca^2+^ stores [43]. Alternatively, extracellular Ca^2+^ may enter the cell through ionotropic receptors (e.g. glutamate receptors) or voltage-gated calcium channels [19]. Given the large amounts of glutamate and GABA released within the SNr, we first characterized the receptors and channels expressed by SNr astrocytic cells, by applying glutamate and GABA (leading to a calcium rise in glial cells since its action on GABA_A_ receptors leads to a membrane depolarization in these cells [44]). Bath applications of glutamate (100 µM) induced a calcium increase in 59.3±8.8% SNr astrocytes (Fig. 4A; *n* = 15 slices, 888 cells). Using the same conditions, GABA (20 µM) induced a calcium rise in 23.5±8.0% SNr astrocytes (Fig. 4C; *n* = 18 slices, 1053 cells).

These agonist-induced calcium responses may reflect a direct effect on astrocytic receptors and/or an indirect effect via neuronal receptors leading to secondary transmitter release. We therefore blocked neuronal transmission with TTX (500 nM) and observed that it did not inhibit the astrocytic calcium response induced by glutamate or GABA suggesting a direct action of these mediators on astrocytic receptors (Fig. 4B and D; *n* = 12 and 9 slices respectively; *p* = 0.32 and 0.21 respectively).

To identify the molecular actors responsible for the induced calcium responses, we applied specific inhibitors of the agonists tested. CNQX (10 µM), an AMPA receptor inhibitor, significantly decreased the glutamate-induced response (55.3±4.0% inhibition; *p*<0.001; *n* = 8 slices, 346 cells). LAP-3 (100 µM), a group I mGluR inhibitor also significantly decreased the glutamate-induced response (31.5±4.6% inhibition; *p* = 0.005; *n* = 11 slices, 552 cells; Fig. 4B), whereas AP-5 (50 µM), a selective NMDAR inhibitor, had no significant effect (9.5±5.1% inhibition; *p* = 0.453; *n* = 4 slices, 130 cells). The combination of these glutamate receptors inhibitors (CNQX 10 µM, LAP-3 100 µM, AP-5 50 µM) produced an additive significant decrease on the glutamate-induced Ca^2+^ response (84.0±7.7% inhibition; *p* = 0.003; *n* = 4 slices, 332 cells; Fig. 4A). We confirmed the lack of response dependent on NMDAR in SNr astrocytes by applying NMDA 100 µM with TTX 500 nM (2.6±1.2% active astrocytes, *n* = 6 slices, 122 cells). Bicuculline (BMI, 20 µM; an inhibitor of GABA_A_ receptors) significantly decreased the GABA-induced Ca^2+^ response (77.2±15.8% inhibition; *p* = 0.02; *n* = 7 slices, 490 cells; Fig. 4D) while 2-hydroxysaclofen 100 µM (an inhibitor of GABA_B_ receptors) had no significant effect (12.1±2.4% inhibition; *p* = 0,948; *n* = 8 slices, 328 cells; Fig. 4D), suggesting that GABA_B_ receptors are poorly expressed by SNr astrocytes.

These pharmacological data provide strong evidence for the expression of AMPA, mGlu and GABA_A_ receptors by SNr astrocytes, all of which being potentially involved in calcium signaling in these cells.

### Contribution of glutamate and GABA to the spontaneous calcium activity of SNr astrocytes

As shown above, the subthalamo-nigral and pallido-nigral pathways were partially preserved in sagittal slices. As neurons are known to be spontaneously active in these nuclei, even in slice preparations [2], [45], SNr activity is constantly modulated by the balance between GABA and glutamate, originating from the GPe and STN, respectively. This balance could also potentially influence astrocytic activity within the SNr, as these cells were found to express the required receptors. As only half of the spontaneous calcium activity of astrocytes is autonomous within SNr, we investigated the relative contribution of glutamatergic and GABAergic influences to the non-autonomous part of spontaneous calcium activity. The application of a combination of glutamate receptors inhibitors (10 µM CNQX, 100 µM LAP-3, 50 µM AP-5) inhibited 63.6±11.1% active astrocytes within the SNr (Fig. 4E and F; *p*<0.001; *n* = 10 slices from 7 rats, 629 cells). Bicuculline 20 µM (BMI) inhibited 47.7±18.8% spontaneous active SNr astrocytes (*p* = 0.003; *n* = 7 slices from 5 rats, 447 cells).

Thus, like SNr neurons, SNr astrocytes display spontaneous activity that is partially dependent on the release of GABA and glutamate within the SNr.

### Modulation of the activity of astrocytes by electrical stimulation of the STN

**Figure 5:**
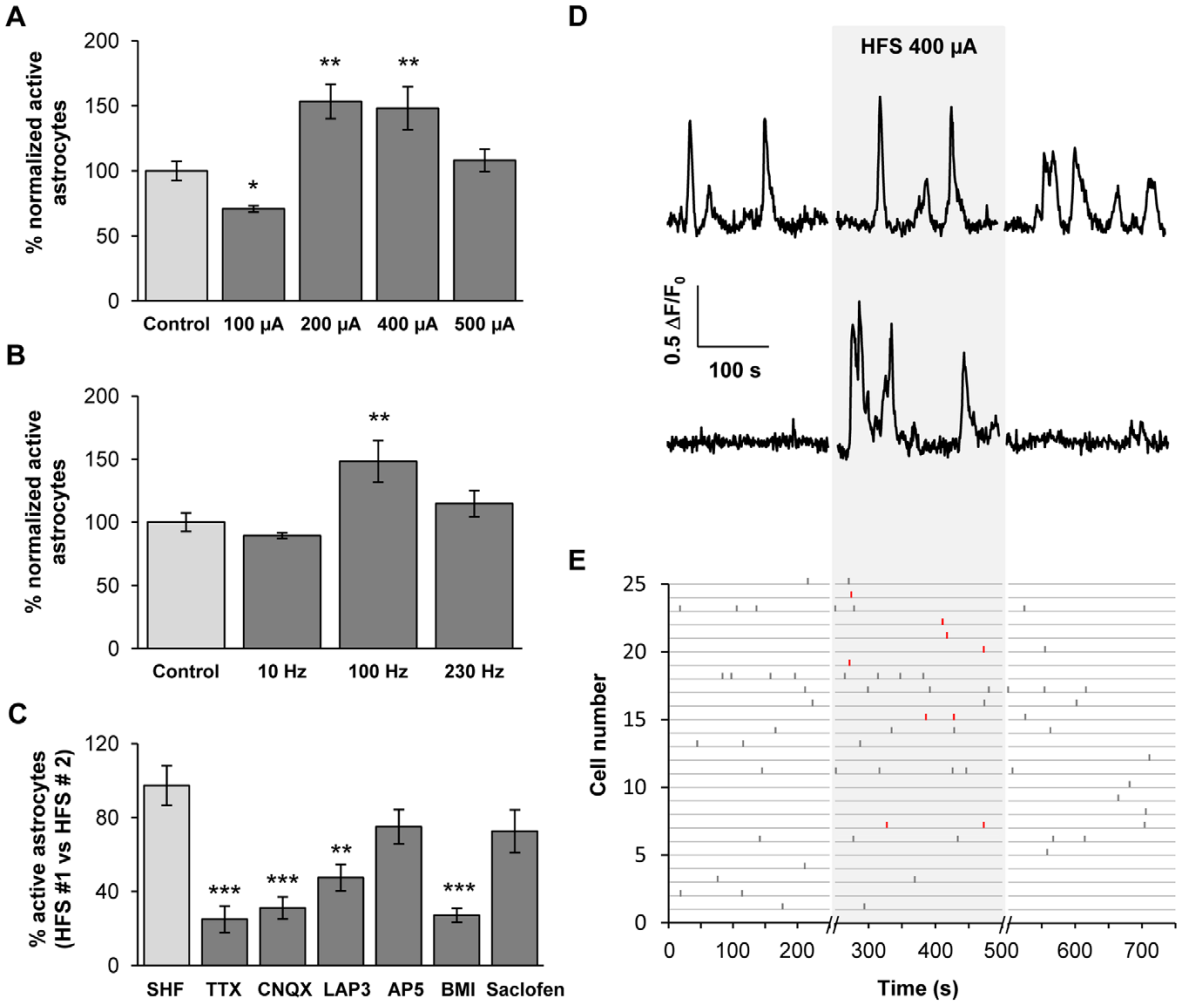
SNr astrocytic calcium activity is modulated by STN electrical stimulation. (**A**) Proportion of loaded astrocytes in the SNr displaying calcium activity during five minutes of STN-HFS (100 Hz) at an intensity of 100 µA (*n* = 14; *p* = 0.011), 200 µA (*n* = 15; *p* = 0.008), 400 µA (*n* = 14; *p* = 0.004) or 500 µA (*n* = 8; *p* = 0.375). (**B**) Proportion of loaded astrocytes in the SNr displaying calcium activity during 5 minutes of STN electrical stimulation at 400 µA with a frequency of 10 Hz (*n* = 15; *p* = 0.896), 100 Hz (*n* = 14; *p* = 0.004) or 230 Hz (*n* = 5; *p* = 0.817). The effect of STN electrical stimulation was normalized with respect to control residual activity. (**C**) Percentage of SNr astrocytes responding to STN electrical stimulation (100 Hz, 200 µA) in the presence of ACSF (SHF; *n* = 9), 500 nM TTX (*n* = 9; *p* = 0.001), 10 µM CNQX (*n* = 9; *p*<0.001), 100 µM LAP-3 (*n* = 8; *p* = 0.01), 50 µM AP-5 (*n* = 13; *p* = 0.121), 20 µM bicuculline (BMI; *n* = 30; *p*<0.001) or 100 µM saclofen (*n* = 7; *p* = 0.266). Inhibitory effects were compared to a first recording under STN-HFS in the same area. *, *p*<0.05; **, *p*<0.01 and ***, *p*<0.001. (**D**) Typical fluorescence variations recorded in two SNr astrocytes before, during (gray background) and after STN-HFS (100 Hz, 400 µA) (**E**) Raster plot of fluorescence peaks detected in active ROIs before, during (gray background) and after STN-HFS (100 Hz, 400 µA), exemplifying that only the number of active cells is enhanced while the frequency in active cells is not affected. Newly active cells during STN-HFS are depicted in red.

STN-HFS is a remarkable tool for alleviating the motor symptoms of advanced Parkinson's disease but the cellular substrates responsible for these promising results remain a matter of debate. STN-HFS may produce its effects by activating the axons of STN neurons, STN afferents or fibers passing close to the stimulation site, leading to the release of glutamate and/or GABA within the SNr [15], [16], [17], [35]. In normal rats, STN-HFS generates concomitant excitatory-inhibitory synaptic currents in SNr neurons by recruiting efferent and passing fibers [34]. Depending on the amplitude and frequency used, electrical stimulation has been shown to differentially impact the glutamate and/or GABA release within SNr [16], [46]. These data raised questions about the effects of STN electrical stimulation on the activity of SNr astrocytes in sagittal slices. We applied bipolar electrical stimulation to the STN during a 5 minute recording period, varying amplitude or frequency parameters. First, using a stimulation frequency of 100 Hz, we varied the amplitude (Fig. 5A). We prior checked that single STN electrical stimulations were effective to induce postsynaptic currents in SNr neurons in our conditions. At 100 µA, STN-HFS triggered a significant decrease in the proportion of active astrocytes in the SNr (70.8±10.6%; *p* = 0.011; *n* = 9 slices from 7 rats, 514 cells). At 200 µA, a significant increase in the proportion of active astrocytes was observed (153.4±42.3%; *p* = 0.008; *n* = 15 slices from 12 rats, 1006 cells). This significant increase also occurred for an amplitude of 400 µA (148.2±38.2%; *p* = 0.004; *n* = 14 slices from 10 rats, 867 cells) but was not observed at an amplitude of 500 µA (108.1±18.7%; *p* = 0.375; *n* = 8 slices from 6 rats, 619 cells). Thus, like SNr neurons, astrocytes were differentially affected by STN-HFS induced imbalance in glutamate/GABA release.

In a second series of experiments, we set the amplitude at 400 µA and varied the frequency (Fig. 5B). At a low frequency (10 Hz), STN electrical stimulation had no effect on the proportion of active astrocytes (89.3±22.5%; *p* = 0.896; *n* = 15 slices from 11 rats, 684 cells). Similarly, at 230 Hz, STN electrical stimulation did not induce any significant effect on the number of active astrocytes (114.7±44.8%; *p* = 0.817; *n* = 5 slices from 4 rats, 183 cells).

Whatever the stimulation parameters used no significant difference in calcium signal duration, frequency or amplitude was observed when electrical stimulation was delivered to the STN. When STN-HFS was applied, the delay of occurrence of the first calcium event in active astrocytes was 110.7±8.0 s (*n* = 9 slices, 121 active cells). This delay was not significantly different from the one measured without STN-HFS (106.1±9.4 s; *p* = 0.537; *n* = 9 slices, 94 active cells). No synchronous activity between active astrocytes was highlighted during STN electrical stimulation. Thus, only the percentage of active astrocytes was affected by STN-HFS and these newly active astrocytes were recruited all along the STN-HFS period (Fig. 5D and E).

At the end of the STN-HFS period, applied at an efficient amplitude (200–400 µA), the proportion of active astrocytes returned to its basal level and no residual effect was observed during a 5 minutes recording post STN-HFS (91.2±12.0% residual activity; *p* = 0,383; *n* = 7 slices from 6 rats, 369 cells; Fig. 5D and E).

When the electrode was placed outside the STN, just to the side of the internal capsule or in a medial position, no significant response was observed within the SNr. Moreover, when TTX (500 nM) was applied locally in the SNr during STN electrical stimulation (100 Hz, 200 µA), we observed a significant inhibition of the SNr astrocytic calcium activity (24.9±7.2% remaining active astrocytes; *p* = 0.001; *n* = 9 slices, 463 cells; Fig. 5C). This residual effect corresponds to the autonomous part of the astrocytic activity. This implies that the observed effect was specifically due to the stimulation of subthalamo-pallido-nigral projections rather than to electrical field diffusion.

Thus, when the parameters used corresponded to those routinely used in parkinsonian patients (with the amplitude adapted to the size of the rat STN [46], [47] *i.e.* 200 to 400 µA), electrical stimulation of the STN induced significant astrocyte activation in the SNr as testified by the increase in the proportion of astrocytes displaying intracellular calcium variations. This activation did not persist when STN-HFS is switched off.

### Involvement of astrocytic AMPA, mGlu and GABA_A_receptors in the increase of SNr astrocytic calcium activity induced by STN-HFS

In order to assess the implication of GABA and glutamate release in the modulation of the astrocytic calcium response triggered by STN electrical stimulation, we locally inhibited these transmitter receptors and compared the effect of STN electrical stimulation (100 Hz, 200 µA) on calcium response in the SNr without inhibitor application. We investigated whether the calcium response was dependent on glutamate by locally applying glutamate receptor inhibitors in the SNr. CNQX (10 µM) decreased the astrocytic calcium response induced by STN-HFS (31.1±6.0% remaining active astrocytes; *p*<0,001; *n* = 9 slices, 505 cells). LAP-3 (100 µM) also significantly decreased the SNr calcium response (47.5±7.2% remaining active astrocytes; *p* = 0.01; *n* = 8 slices, 352 cells), whereas AP-5 (50 µM) had no significant effect (75.03±9.2% remaining active astrocytes; *p* = 0,121; *n* = 13 slices, 487 cells). Application of a cocktail of glutamate receptor inhibitors (CNQX 10 µM, LAP-3 100 µM, AP-5 50 µM) to the SNr induced a significant decrease in the number of responding cells (34.2±4.9% remaining active astrocytes; *p*<0.001; *n* = 11 slices, 844 cells), in the same range as CNQX alone. These data suggest that an increase in glutamate release within the SNr during STN electrical stimulation is partly responsible for the astrocytic calcium response.

Local application of bicuculline (BMI, 20 µM) to the SNr significantly decreased the SNr astrocytic calcium response to STN electrical stimulation (27.1±3.8% remaining active astrocytes; *p*<0.001; *n* = 24 slices, 1495 cells; Fig. 5C), indicating that GABA is also involved in the STN-HFS effect on astrocytes within the SNr through GABA_A_R activation. In the same conditions, 2-hydroxysaclofen (100 µM) failed to significantly inhibit the STN electrical stimulation effect (72.6±11.6% remaining active astrocytes; *p* = 0,266; *n* = 7 slices, 380 cells), confirming that GABA_B_Rs are not involved in the SNr astrocytic response. Concomitant applications of glutamate receptor inhibitors and BMI had no additive inhibitory effect compared to those obtained with each group of inhibitor (not shown). These results suggest that glutamate and GABA are both involved in the astrocytic calcium response induced by STN-HFS. Astrocytic AMPAR, mGluR and GABA_A_R are involved in this activity increase.

## Discussion

In this study, we used calcium imaging to monitor SNr astrocytic calcium activity and the effects of modulating some of the basal ganglia networks that are known to influence the regulation of the SNr output activity. We took advantage of the sagittal brain slice preparation that preserved the pace-maker activity of the STN-GPe-SNr loops to modify its activity and investigate the impact of STN electrical stimulation on the activity of SNr astrocytes. SNr astrocytes displayed spontaneous calcium activity in basal physiological conditions. About half (48%) of this activity was found to be autonomous (*i.e.* independent of neuronal activity) while the other half is dependent on the spontaneous release of glutamate and GABA, probably under the control of the STN- SNr and GPe-SNr pathways. Changes of the activity of STN-GPe-SNr loops (induced by STN electrical stimulation) affected this astrocytic calcium activity in a consistent way to the amount of current delivered to the STN. Astrocytic AMPAR, mGluR and GABA_A_R are involved in the specific response of SNr astrocytes when STN is electrically stimulated. Thus, in physiologically preserved active loops, astrocyte activity is affected by glutamate/GABA release as is SNr neuronal output activity.

### Technical considerations and validity of the model

The excitability of astrocytes is mediated by increases in the intracellular Ca^2+^ concentration [22]. Calcium imaging provides readout of this cell excitation, monitoring the activities of dozens of cells simultaneously. Its use on brain slices makes it possible to study cells in a physiological context, preserving some networks and environmental background. Astrocytes represent the major cell population in the SNr but their role remains unclear. In our loading conditions, few neurons were loaded in the SNr allowing specific study of the activity of astrocytes. However, we showed that most of the SNr neuronal cells in the brain slice were functional even if they were not loaded.

Sulforhodamine stainings revealed that more than 98% of SNr recorded cells were astrocytes, meaning that involvement of other glial cells may be considered insignificant.

The sagittal slice allows study of the communication between astrocytes and neurons in an integrated loop endowed with spontaneous pace-maker activity. A major point is that we can modulate the SNr activity by electrically stimulating a distant nucleus (*e.g.* STN). These nuclei (STN, GPe and SNr) are of major importance within the basal ganglia network in physiological or pathological conditions (*e.g.* Parkinson disease) and are at the heart of the most famous example of a functional restoration of the network activity by STN-HFS. Thus, this model will be very useful for deciphering the nature of neuron-astrocyte communication within the SNr, in physiopathological conditions and under STN-HFS. More often, non physiological or direct stimulations were used in astrocyte studies in other brain areas [48], [49], [50]. Modulating STN-GPe-SNr loops with STN-HFS allows to interact in a physiological way with SNr astrocytes.

### Evoked and autonomous spontaneous astrocytic calcium activity

We showed that only 48% of astrocytic calcium activity in the SNr is autonomous, *i.e.* independent of neuronal activity. In other brain structures such as the hippocampus [48], [51] and somatosensory cortex [52], astrocytic calcium activity is known to be totally independent of neuronal activity and is therefore considered autonomous. Indeed, in these structures, there is no tonic spontaneous release of neurotransmitters at rest. The spontaneous pace-maker activity of STN-GPe-SNr loops and the continual release of glutamate and GABA within the SNr, probably account for the distinctive nature of the spontaneous calcium activity in SNr astrocytes, fitting with their environment and sensing the neurotransmitter released. Thus, the existence of two different modes of spontaneous Ca^2+^ signaling within astrocytes, *i.e.* autonomous and activity-evoked, should have specific physiological roles fitting with astrocyte function in SNr [53].

The evoked part of spontaneous calcium activity is dependent, at least, on glutamate and GABA release within the SNr. GABAergic pallidal activity, glutamatergic STN activity and/or GABAergic intranigral activity may potentially contribute to this evoked basal glial calcium activity. *In vivo*, neurochemical data have shown that, within intact SNr, the extracellular glutamate concentration is 16 times higher than GABA [54]. Indeed, the proportion of glutamate-responding astrocytes is significantly higher than the proportion of GABA-responding astrocytes (59.3±8.8% versus 23.5±8.0%; *p*<0,001). This heterogeneity in astrocytic sensitivity might depend on their proximity to intranigral axon collaterals, GPe-SNr or STN-SNr projections, as described in hippocampus [55]. The major input to GABAergic SNr neurons arises from STN whereas GABAergic projections from GPe are fewer [56], [57]. This particular astrocyte sensitivity will be further explored in pathological situations where the GABA/glutamate balance is disrupted.

Moreover, transmitters such as serotonin, endocannabinoid and dopamine could also be involved in part of this evoked spontaneous activity. We will further explore the contribution of these pathways to neuron-astrocyte communication within SNr.

### Imbalance of the STN-GPe-SNr loops through STN electrical stimulation

Given the importance of the GABA/glutamate balance in the SNr output activity [13], [14], [15], studies on the effect of this balance on glial cell-dependent neuromodulation are of great interest. The efficacy of STN-HFS for alleviating Parkinson's disease symptoms has highlighted the value of treatment strategies bypassing the dopamine system to restore balance in the basal ganglia motor circuitry. *In vivo*, neurochemical data have shown that electrical stimulation of STN at clinical parameters induces an increase of both glutamate and GABA contents in the SNr of intact rats [15]. These data have been confirmed by electrophysiological studies performed in rats [13], [34] and monkeys [58] demonstrating a concomitant excitatory and inhibitory effect of STN-HFS on SNr neuronal activity mediated by glutamate and GABA action. These complex effects are known to depend on current intensity [34], [46] and frequency [11], [16]. *In vivo*, a weak current intensity (<60 µA) did not affect extracellular glutamate and GABA levels in SNr of intact rats,[46]. In our model, we showed that a weak intensity (100 µA) significantly decreased the proportion of active astrocytes in the SNr. This could be due to an imposed decrease of the activity pattern of STN neurons as reported in rat brain slices at such a low intensity [11]. On the contrary, when intensities are close to clinical parameters, STN-HFS is known to impose a new activity pattern on STN neurons [11] together with activation of pallidonigral fibers and the intranigral axon collaterals of SNr cells [13] leading to concomitant glutamate and GABA release in SNr during the stimulation period [46]. In agreement with these data, we found that the proportion of active astrocytes significantly increased at 200 and 400 µA. This increase was due to the activation of astrocytic AMPA, mGlu and GABA_A_ receptors. Thus, there is a window of STN-HFS intensity that enhances calcium activity in astrocytes through an increase of GABA and glutamate release. Higher intensity (500 µA) had no effect on the astrocyte calcium activity even though an effect on SNr neuronal activity has been reported in rat brain slices [34].

In the same way, it has been shown that low frequency (10 Hz) STN electrical stimulation did not modify ongoing STN activity [11] and did not affect glutamate and GABA levels within SNr in intact rats [33]. Concordantly, we showed that low frequency (10 Hz - 400 µA) STN electrical stimulation had no effect on the proportion of active astrocytes. At 350 Hz, it has been reported that only GABA increased, due to a differential recruitment of STN and GPe fibers [16]. In our model, at 230 Hz (we could not go further without damaging the slice) there is no effect on the proportion of active astrocytes. It is possible that the frequency threshold to recruit only GPe fibers was not reached. It could also be due to a differential distribution of nearby GABAergic projections from GPe *vs* intranigral axon collaterals or to regionalization of GABA_A_R within astrocytes leading to a lesser sensitivity of SNr astrocytes to GABA release from GPe.

Altogether these results reveal that modification of GABA/glutamate release strongly affect not only neurons but also the activity of the astrocytic compartment. Only the proportion of active astrocytes is enhanced suggesting a supplemental recruitment of astrocytes, potentially close to activated synapses. This recruitment is concomitant with STN-HFS and did not persist after the STN-HFS has been switched off. Thus, the additional calcium signaling may activate specific astrocytic pathways suggesting functional consequences on overall activity of SNr.

### Functional relevance

Calcium is considered as a key second messenger in astrocyte-neuron communication. Astrocytes use a different language, a different way of getting input and output, on a totally different timescale from neurons but superimposed with it. The functional consequence on the information leaving the SNr might be complex to decipher. Modulating the astrocytic calcium activity in a physiological loop would allow the study of its impact on SNr neuronal signaling. Astrocytes are involved in various and ubiquitous functions within the brain, some of them being calcium-dependent [39]. Indeed, astrocytic calcium activity has been involved in short-term and long-term plasticity [59] but a better understanding of calcium dynamics, signaling and gliotransmitter release is necessary to underline calcium as the key second messenger in astrocyte-neuron communication and neuronal plasticity in Parkinson disease. Few studies have focused on the involvement of astrocytes in the basal ganglia network activity. In normal rats, it has been shown that astrocytes, via the uptake of neurotransmitters, increase the strength of filtering operated through striatal medium spiny neurons [60]. In parkinsonian monkeys, a significant expansion of the astrocyte coverage has been described in glutamatergic striatal synapses providing evidence for compensatory changes that affect both neuronal and astrocytic elements in striatum, suggesting an active role in transmitter homeostasis [61]. Optogenetic studies, performed in hemiparkinsonian rats, reported that light stimulation of STN astrocytes inhibited neuronal firing in STN although no motor consequences were observed [62]. Thus, it becomes crucial to fully take into account and investigate the contribution of astrocytes in the physiology and pathophysiology of the basal ganglia output structures. Combining calcium imaging, patch-clamp experiments and physiological modification of the STN-GPe-SNr loop activity will allow us to go further in this field.

### Conclusion

The originality of this study lies in its novel approach, complementary to the traditional research route focusing mainly on the influence of neurons within pathophysiological mechanisms. More specifically, it brings new insight concerning the role of astrocytes in deep brain stimulation mechanisms. STN-HFS affecting SNr astrocytic calcium activity suggests a potential functional role for astrocytes in pathophysiological processes concerning this basal ganglia output structure. The glutamate/GABA balance is already known to be of major importance in the regulation of SNr neuronal activity, mainly through the drastic effects of its deregulation. For the first time, we show that modification of glutamate and GABA release also has a strong impact on astrocyte activity.The actual knowledge on neuron-astrocyte communication prods us into hypothesize that astrocytes could be effective partners in the modulation of SNr neuronal activity and in counteracting abnormal neuronal activity within the basal ganglia network. The next step will be to further study the impact of the astrocytic calcium activity on the SNr output neuron activity either in physiological or in dopamine denervation conditions.

## Materials and Methods

### Slice preparation

Sagittal slices containing the rostral midbrain (300 µm thick) were prepared from young male Sprague-Dawley rats (postnatal days 17–21; Janvier, Le Genest St Isle, France). In compliance with the European Community Council Directive of November 24, 1986 (86/609/EEC), research involving animals has been authorized by the Direction Départementale de la Protection des Populations, Préfecture de l'Isère, France (M. Albrieux, PhD, permit number 38 10 22; S. Boisseau, PhD, permit number 38 09 35). Moreover, all animal procedures were carried out in accordance with the French guidelines on the use of living animals in scientific investigations with the approval of the “Grenoble Institute of Neurosciences ethical committee” (agreement number 004). Every effort was made to minimize the number of animals used and their suffering during the experimental procedure. Rats were killed by decapitation and their brains were removed rapidly and cut into slices with a vibratome VT1000S (Leica, Wetzlar, Germany) in cold low Ca^2+^-high Mg^2+^ artificial cerebral spinal fluid (ACSF) containing: 126 mM NaCl, 2.5 mM KCl, 7 mM MgCl_2_, 0.5 mM CaCl_2_, 1.2 mM NaH_2_PO_4_, 25 mM NaHCO_3_, 11 mM D-glucose, bubbled with 95% O_2_/5% CO_2_. Sagittal slices containing the GPe, the STN and the SNr were placed in ACSF (126 mM NaCl, 2.5 mM KCl, 1.2 mM MgSO_4_, 2.5 mM CaCl_2_, 1.2 mM NaH_2_PO_4_, 25 mM NaHCO_3_, 11 mM D-glucose) saturated with 95% O_2_/5% CO_2_ and supplemented with 1 mM sodium pyruvate at room temperature, for a recovery period [63].

### Projection tracing

We checked the integrity of the STN-SNr and GPe-SNr pathways on the sagittal slices by injecting DiI into these nuclei. We fixed acute slices in 4% paraformaldehyde solution (Electron Microscopy Science, Hatfield, Pennsylvania, USA) overnight at room temperature. We then placed crystals of DiI (1,1′-dioctadecyl-3,3,3′,3′-tetramethylindocarbocyanine perchlorate, Molecular Probes, Eugene, Oregon, USA) on the STN or the SNr for anterograde or retrograde labeling. Slices were washed twice, for 10 minutes each in 0.1 M PB (0.1 M potassium phosphate and 0.019 M sodium phosphate; pH 7.2), then kept in the dark at room temperature for 4 weeks [64].

### Cell viability

In some experiments, cell viability was estimated at various stages of the slicing, loading and recording protocol, by propidium iodide staining (0.75 µM; Sigma-Aldrich) for 1 minute. The slices tested had previously been incubated with Hoechst 33342 (5 µM; Molecular Probes) for 30 minutes in the dark. Images were acquired with an upright compound microscope (Eclipse E600 FN, Nikon, Paris, France) equipped with a water immersion 20× objective (NA 0.5) and a confocal head (confocal C1 head, Nikon, Paris, France).

### Immunostaining

Postnatal day 20 rats (*n* = 6) were anesthetized with chloral hydrate (0.5 ml/100 g) and killed by transcardiac perfusion with 10 ml phosphate buffer saline (PBS, 0.1 M) followed by 35 ml 4% paraformaldehyde (Electron Microscopy Science, Hatfield, Pennsylvania, USA). Brains were quickly removed from the skull after the perfusion and postfixed in 4% paraformaldehyde at 4°C overnight. Brains were then rinsed in 20% sucrose (Sigma) for 48 h, frozen in dry ice (−40°C) and stored at −80°C until use. Sagittal tissue sections at the SNr level (20 µm) were cut on a cryostat (HM 500 M, Microm, Francheville, France) and collected in antifreeze solution. Floating sections were washed in TBS buffer (0.1 M Tris Base, 0.15 M NaCl) and blocked by incubation with 3% normal goat serum (NGS, Interchim, Montluçon, France) in TBS-TX (0.1 M Tris Base, 0.15 M NaCl, 0.2% Triton X-100) for 30 minutes (dilution/blocking buffer). Tissue sections were then incubated overnight at 4°C with following antibodies: neuron-specific anti-neuronal nuclei antibody [NeuN, Chemicon (Chemicon Europe, Hants, UK), 1∶500 in TBS buffer with 1% normal goat serum (dilution buffer)], anti-S100B antibody (Sigma), 1∶100 in dilution buffer, anti-glial fibrillary acidic protein (GFAP) antibody [Dako (Dako, Glostrup Denmark)], 1∶1000 in dilution buffer, microglial cell-specific anti-CD11b antibody [Ox-42, AbCys (AbCys, Paris, France) 1∶100 in dilution buffer], oligodendrocyte lineage-specific anti-Olig 2 antibody [Millipore (Millipore, Billerica, MA, USA), 1∶100 in dilution buffer], anti-NG2 antibody (Millipore, 1∶100 in dilution buffer). Tissue sections were washed in TBS-TX and incubated for 2 h at room temperature with the appropriate Alexa Fluor 488-conjugated (Molecular Probes) or cyanin 3-conjugated (Jackson ImmunoResearch Laboratories) secondary antibody, in dilution buffer (1∶1000). Sections were washed in TBS-TX, incubated with TO-PRO-3 iodide (InVitrogen, 1∶1000) for 20 minutes at room temperature for nuclear staining, washed in TBS and mounted in Vectashield mounting medium (Vector Laboratories Inc, Burlingame, CA, USA). Images were acquired with a TCS-SP2 confocal setup (Leica, Deerfield, IL, USA). For illustrations, images were treated (brightness and contrast adjustments) and merged with Adobe Photoshop CS.

Following calcium imaging, some acute horizontal brain slices (300 µm thickness) loaded with Fluo-4 were fixed by incubation in 4% paraformaldehyde and 10 mg/ml EDC (1-ethyl-3-(3-dimethylaminopropyl) carbodiimide, Sigma) in PBS (31.6 mM NaH_2_PO4, 68.4 mM Na_2_PO_4_, 150 mM NaCl) for 1 h at room temperature, in the dark. EDC fixes calcium chelators, such as Fluo-4 [65]. Immunostaining was then performed as described above.

### Sulforhodamine staining

Sulforhodamine 101 sodium salt (Sigma-Aldrich) was injected intravenously at a volume of 100 µl using a concentration of 10 mg/ml in saline solution. Animals were briefly anesthetized using isoflurane in a 70% air, 30% O_2_ gas mixture for the intravenous injection (100 µl/50 g body weight). After 2 h, the rats were decapitated and their brains were rapidly removed. Acute sagittal brain slices were sectioned as described above.

When required, slices were loaded with Hoechst 33342 (5 µM; Molecular Probes) for 30 minutes to stain cell nuclei. Stained slices were then placed in a constantly perfused chamber (ACSF bubbled with 95% O_2_/5% CO_2_) on the stage of a two-photon laser scanning microscope (TPLSM 7 MP, Zeiss, Germany) equipped with a 20× water-immersion objective (NA 1.0; Zeiss) and images were acquired with ZEN 2009 software. Laser excitation at 800–900 nm was carried out with a Ti:Sapphire laser system (Chameleon vision II; Coherent, UK). The excitation wavelengths used for the sulforhodamine dye and Hoechst were respectively 880 nm and 750 nm.

In some experiments, slices were loaded with Fluo-4 AM (5 µM; Molecular Probes) for 30 minutes and placed in a constantly perfused chamber (ACSF bubbled with 95% O_2_/5% CO_2_) on the stage of an upright compound microscope (Eclipse E600 FN, Nikon, Paris, France) equipped with a water immersion 20× objective (NA 0.5) and a confocal head (confocal C1 head, Nikon, Paris, France). SR101 was excited at 543 nm and emission was filtered with a 605±75 nm filter. Fluo-4 AM was excited with an argon laser at 488 nm and emission was filtered with a 515±15 nm filter.

### Stereological quantification

Acquisitions were performed with a translating platform with motorized crossed roller stages (8 fields of 425 µm×425 µm for each SNr area). Image processing was performed with ImageJ software [66]. Multistack (40 slices at 2 µm spacing) mosaics were reconstructed with the “3D stitching” plugin of ImageJ [67]. Quantitative analysis was based on a two step procedure. First, raw images were segmented by adaptive thresholding (Bernsen) to facilitate automated cell detection. The result was then analyzed with the “3D object counter” plugin [68] in ImageJ. The results are presented as mean ± SEM.

### Calcium imaging on brain slices

Brain slices were loaded with the calcium indicator dye Fluo-4 by immersion for 30 minutes at 35°C in a bath containing 5 µM Fluo-4 AM (Molecular Probes, Eugene, Oregon, USA), 0.005% Pluronic acid F-127 (Molecular Probes), 0.00025% Cremophor EL (Sigma-Aldrich) and 0.05% DMSO (dimethyl sulfoxide, Sigma-Aldrich) in ACSF. The loading chamber was continuously oxygenated with 95% O_2_/5% CO_2_. Slices were then placed in ACSF saturated with 95% O2/5% CO2 and supplemented with 1 mM sodium pyruvate, at room temperature for at least 45 minutes before imaging. Next, slices were placed in a constantly perfused chamber (ACSF bubbled with 95% O_2_/5% CO_2_) on the stage of an upright compound microscope (Eclipse E600 FN, Nikon, Paris, France) equipped with a water immersion 20× objective (NA 0.5) and a confocal head (confocal C1 head, Nikon, Paris, France). Experiments were initially performed at both 35°C or at room temperature. No difference in the percentage of activated cells was observed so, for practical reasons, most of the experiments were then performed at room temperature. Excitation was achieved with light at 488 nm from an argon laser and emission was filtered with a 515±15 nm filter. Images were acquired with EZ-C1 software (Nikon, Paris, France), in a 12-bit encoded format. Images were taken at 800 ms intervals in a single confocal plane over a period of 5 minutes.

Pharmacological agonists, such as glutamate, NMDA and GABA (Sigma-Aldrich), or inhibitors, such as tetrodotoxin (TTX; Latoxan, Valence, France), thapsigargin (Molecular Probes), CNQX, L(+)-2-amino-3-phosphonopropionic acid (L-AP3), D(−)-2-amino-5-phosphonopentanoic acid (AP-5), bicuculline (BMI) and 2-hydroxysaclofen (Sigma-Aldrich), were perfused in the bath. The inhibitory effects of antagonists on spontaneous calcium activity were assessed by comparison to a first recording of calcium activity performed in the same area. In these conditions, the percentage of residual active cells in a control experiment was not significantly different in the second recording (79.5±7.4%, *p* = 0.183, *n* = 18 slices from 11 rats, 1077 cells) and there was also no significant difference in the calcium signal parameters (amplitude, duration, frequency). The inhibitory effect of all tested antagonists was assessed by comparison with this control experiment and normalization. The evoked effect of glutamate and GABA on astrocyte calcium activity and the inhibitory effect of antagonist on exogenous glutamate and GABA applications were assessed by analyzing the cells on addition of the agonist to the bath and in the 10 images following agonist addition. The inhibitory effects of antagonists on STN-HFS induced activity were compared versus a control group of HFS where two successive stimulations were applied to the slice. In that case, the HFS #2 represented 97.3±11.6% of the HFS #1 response (*p* = 0,570; *n* = 9 slices from 8 rats, 754 cells). In these experiments, inhibitors were applied locally in order to affect the SNr but not the STN or GPe.

### Quantification and statistical analysis

CalSignal software was used to detect cells and measure their intracellular Ca^2+^ activity [69]. This software can automatically detect and track hundreds of cells per slice and carries out statistical analysis for numerous parameters, analyzing the fluorescence signal F within each region of interest. F_0_ was calculated for each region of interest (ROI) on the recording period or, when an agonist was applied, it was calculated on the period before the drug application. Based on the ΔF/F_0_ ratios, significant fluorescence variations were detected and their amplitude and duration were calculated. These data were exported to SigmaStat software (Systat) for statistical analysis. All results are presented as mean ± SEM. When inhibitors were applied, each slice was its own control since we compared the effect of the pharmacological agent tested on spontaneous calcium activity with an initial recording in the same area during the same duration before treatment. Statistical analyses were carried out with Mann-Whitney-Wilcoxon tests with significance levels of *, *p*<0.05; **, *p*<0.01 and ***, *p*<0.001.

We performed cross-correlation analysis to estimate synchrony between cells. The cross-correlations between the calcium signals of couple of cells were calculated at time lags varying between −3 and +3 s using the “crosscoef” function in Matlab. When the cross-correlation coefficient exceeded 0.7, the cell couples were considered as synchronous [48].

We used young rats to optimize Fluo-4 loading. We therefore checked the possible age-dependence of the recorded response. We found no significant differences (Kruskal-Wallis analysis) in either the percentage of cells displaying spontaneous calcium activity (P17: 33.1±4.7%; P18: 26.7±3.1%; P19: 35.7±5.7%; P20: 21.0±4.7%; *p* = 0,232), or the percentage of cells responding to electrical (*p* = 0,595) or chemical stimuli (Glutamate: *p* = 0,165; GABA: *p* = 0,267) from P17 to P21, so results were pooled for analysis.

### Electrical stimulation procedures

A concentric bipolar stimulation electrode (platinium/iridium; Frederick Haer & Co., USA) was positioned in the middle of the STN within the brain slice. The electrical stimulation consisted of multiple rectangular pulses (0.1 ms duration; 10,100 or 230 Hz) of constant current (100 to 500 µA) applied during the 5 minute recording period. These parameters correspond to those routinely used in parkinsonian patients, but the amplitude was adapted to the size of the rat STN [46], [47]. At intensities above 600 µA at 100 Hz or 400 µA at 250 Hz, stimulation resulted in tissue damage (burn and lesion). As for the application of inhibitors described above, the impact of STN electrical stimulation on astrocytic calcium activity parameters was determined by comparison with an initial recording of the same area. The positive or negative effect of STN-HFS was assessed by comparison with the previous control experiment and normalization.

## Supporting Information

Figure S1 **S100B and GFAP immunostainings in the SNr.** (**A**) Two-photon multistack (9 slices at 2 µm spacing) mosaic reconstruction of S100B immunostaining in the SNr of a fixed brain slice. Antero-posterior (AP) and medio-lateral (ML) orientations are shown in the upper right part of the figure. (**B**) Confocal images of S100B (green) and GFAP (red) immunostained cells in the SNr of a sagittal section of rat brain. Merged image showing the proportion of S100B and GFAP positive cells (right panel). Nuclei are identified by TO-PRO staining (blue). Arrow indicates a GFAP positive cell that is S100B negative and arrowheads show several S100B-positive cells that are GFAP negative.
(TIF)

## References

[1] Hikosaka O (2007). GABAergic output of the basal ganglia.. Prog Brain Res.

[2] Richards CD, Shiroyama T, Kitai ST (1997). Electrophysiological and immunocytochemical characterization of GABA and dopamine neurons in the substantia nigra of the rat.. Neuroscience.

[3] DeLong MR (1990). Primate models of movement disorders of basal ganglia origin.. Trends Neurosci.

[4] Plenz D, Kital ST (1999). A basal ganglia pacemaker formed by the subthalamic nucleus and external globus pallidus.. Nature.

[5] Kitai ST, Deniau JM (1981). Cortical inputs to the subthalamus: intracellular analysis.. Brain Res.

[6] Smith Y, Bevan MD, Shink E, Bolam JP (1998). Microcircuitry of the direct and indirect pathways of the basal ganglia.. Neuroscience.

[7] Miller WC, DeLong MR (1988). Parkinsonian symptomatology. An anatomical and physiological analysis.. Ann N Y Acad Sci.

[8] Bergman H, Wichmann T, Karmon B, DeLong MR (1994). The primate subthalamic nucleus. II. Neuronal activity in the MPTP model of parkinsonism.. J Neurophysiol.

[9] Benazzouz A, Piallat B, Pollak P, Benabid AL (1995). Responses of substantia nigra pars reticulata and globus pallidus complex to high frequency stimulation of the subthalamic nucleus in rats: electrophysiological data.. Neurosci Lett.

[10] Beurrier C, Bioulac B, Audin J, Hammond C (2001). High-frequency stimulation produces a transient blockade of voltage-gated currents in subthalamic neurons.. J Neurophysiol.

[11] Garcia L, Audin J, D'Alessandro G, Bioulac B, Hammond C (2003). Dual effect of high-frequency stimulation on subthalamic neuron activity.. J Neurosci.

[12] Benazzouz A, Hallett M (2000). Mechanism of action of deep brain stimulation.. Neurology.

[13] Maurice N, Thierry AM, Glowinski J, Deniau JM (2003). Spontaneous and evoked activity of substantia nigra pars reticulata neurons during high-frequency stimulation of the subthalamic nucleus.. J Neurosci.

[14] Degos B, Deniau JM, Thierry AM, Glowinski J, Pezard L (2005). Neuroleptic-induced catalepsy: electrophysiological mechanisms of functional recovery induced by high-frequency stimulation of the subthalamic nucleus.. J Neurosci.

[15] Windels F, Bruet N, Poupard A, Urbain N, Chouvet G (2000). Effects of high frequency stimulation of subthalamic nucleus on extracellular glutamate and GABA in substantia nigra and globus pallidus in the normal rat.. Eur J Neurosci.

[16] Windels F, Bruet N, Poupard A, Feuerstein C, Bertrand A (2003). Influence of the frequency parameter on extracellular glutamate and gamma-aminobutyric acid in substantia nigra and globus pallidus during electrical stimulation of subthalamic nucleus in rats.. J Neurosci Res.

[17] Dostrovsky JO, Lozano AM (2002). Mechanisms of deep brain stimulation.. Mov Disord.

[18] Deniau JM, Mailly P, Maurice N, Charpier S (2007). The pars reticulata of the substantia nigra: a window to basal ganglia output.. Prog Brain Res.

[19] Verkhratsky A, Steinhauser C (2000). Ion channels in glial cells.. Brain Res Brain Res Rev.

[20] Haydon PG (2001). GLIA: listening and talking to the synapse.. Nat Rev Neurosci.

[21] Perea G, Navarrete M, Araque A (2009). Tripartite synapses: astrocytes process and control synaptic information.. Trends Neurosci.

[22] Perea G, Araque A (2005). Glial calcium signaling and neuron-glia communication.. Cell Calcium.

[23] Auld DS, Robitaille R (2003). Glial cells and neurotransmission: an inclusive view of synaptic function.. Neuron.

[24] Wang X, Lou N, Xu Q, Tian GF, Peng WG (2006). Astrocytic Ca2+ signaling evoked by sensory stimulation in vivo.. Nat Neurosci.

[25] Porter JT, McCarthy KD (1996). Hippocampal astrocytes in situ respond to glutamate released from synaptic terminals.. J Neurosci.

[26] Fiacco TA, Agulhon C, McCarthy KD (2009). Sorting out astrocyte physiology from pharmacology.. Annu Rev Pharmacol Toxicol.

[27] Schell MJ, Molliver ME, Snyder SH (1995). D-serine, an endogenous synaptic modulator: localization to astrocytes and glutamate-stimulated release.. Proc Natl Acad Sci U S A.

[28] Panatier A, Theodosis DT, Mothet JP, Touquet B, Pollegioni L (2006). Glia-derived D-serine controls NMDA receptor activity and synaptic memory.. Cell.

[29] Parri HR, Gould TM, Crunelli V (2001). Spontaneous astrocytic Ca2+ oscillations in situ drive NMDAR-mediated neuronal excitation.. Nat Neurosci.

[30] Parri R, Crunelli V (2003). An astrocyte bridge from synapse to blood flow.. Nat Neurosci.

[31] Fiacco TA, McCarthy KD (2004). Intracellular astrocyte calcium waves in situ increase the frequency of spontaneous AMPA receptor currents in CA1 pyramidal neurons.. J Neurosci.

[32] Araque A, Carmignoto G, Haydon PG (2001). Dynamic signaling between astrocytes and neurons.. Annu Rev Physiol.

[33] Paxinos G, Watson C (1998). The rat brain in stereotaxic coordinates.

[34] Bosch C, Degos B, Deniau JM, Venance L (2011). Subthalamic nucleus high frequency stimulation generates a concomitant synaptic excitation-inhibition in substantia nigra pars reticulata.. J Physiol.

[35] Deniau JM, Degos B, Bosch C, Maurice N (2010). Deep brain stimulation mechanisms: beyond the concept of local functional inhibition.. Eur J Neurosci.

[36] Nair-Roberts RG, Chatelain-Badie SD, Benson E, White-Cooper H, Bolam JP (2008). Stereological estimates of dopaminergic, GABAergic and glutamatergic neurons in the ventral tegmental area, substantia nigra and retrorubral field in the rat.. Neuroscience.

[37] Parent A (1990). Extrinsic connections of the basal ganglia.. Trends Neurosci.

[38] Hewett JA (2009). Determinants of regional and local diversity within the astroglial lineage of the normal central nervous system.. J Neurochem.

[39] Wang DD, Bordey A (2008). The astrocyte odyssey.. Prog Neurobiol.

[40] Verant P, Ricard C, Serduc R, Vial JC, van der Sanden B (2008). In vivo staining of neocortical astrocytes via the cerebral microcirculation using sulforhodamine B.. J Biomed Opt.

[41] Appaix F, Girod S, Boisseau S, Romer J, Vial JC (2012). Specific in vivo staining of astrocytes in the whole brain after intravenous injection of sulforhodamine dyes.. PLoS One.

[42] Ikegaya Y, Le Bon-Jego M, Yuste R (2005). Large-scale imaging of cortical network activity with calcium indicators.. Neurosci Res.

[43] Verkhratsky A, Orkand RK, Kettenmann H (1998). Glial calcium: homeostasis and signaling function.. Physiol Rev.

[44] Kettenmann H, Schachner M (1985). Pharmacological properties of gamma-aminobutyric acid-, glutamate-, and aspartate-induced depolarizations in cultured astrocytes.. J Neurosci.

[45] Bevan MD, Magill PJ, Terman D, Bolam JP, Wilson CJ (2002). Move to the rhythm: oscillations in the subthalamic nucleus-external globus pallidus network.. Trends Neurosci.

[46] Boulet S, Lacombe E, Carcenac C, Feuerstein C, Sgambato-Faure V (2006). Subthalamic stimulation-induced forelimb dyskinesias are linked to an increase in glutamate levels in the substantia nigra pars reticulata.. J Neurosci.

[47] Shen KZ, Johnson SW (2006). Subthalamic stimulation evokes complex EPSCs in the rat substantia nigra pars reticulata in vitro.. J Physiol.

[48] Sasaki T, Kuga N, Namiki S, Matsuki N, Ikegaya Y (2011). Locally Synchronized Astrocytes.. Cereb Cortex.

[49] Bernardinelli Y, Salmon C, Jones EV, Farmer WT, Stellwagen D Astrocytes display complex and localized calcium responses to single-neuron stimulation in the hippocampus.. J Neurosci.

[50] Schummers J, Yu H, Sur M (2008). Tuned responses of astrocytes and their influence on hemodynamic signals in the visual cortex.. Science.

[51] Aguado F, Espinosa-Parrilla JF, Carmona MA, Soriano E (2002). Neuronal activity regulates correlated network properties of spontaneous calcium transients in astrocytes in situ.. J Neurosci.

[52] Takata N, Hirase H (2008). Cortical layer 1 and layer 2/3 astrocytes exhibit distinct calcium dynamics in vivo.. PLoS One.

[53] Fellin T (2009). Communication between neurons and astrocytes: relevance to the modulation of synaptic and network activity.. J Neurochem.

[54] Windels F, Carcenac C, Poupard A, Savasta M (2005). Pallidal origin of GABA release within the substantia nigra pars reticulata during high-frequency stimulation of the subthalamic nucleus.. J Neurosci.

[55] Perea G, Araque A (2005). Properties of synaptically evoked astrocyte calcium signal reveal synaptic information processing by astrocytes.. J Neurosci.

[56] von Krosigk M, Smith Y, Bolam JP, Smith AD (1992). Synaptic organization of GABAergic inputs from the striatum and the globus pallidus onto neurons in the substantia nigra and retrorubral field which project to the medullary reticular formation.. Neuroscience.

[57] Groenewegen HJ, Berendse HW (1990). Connections of the subthalamic nucleus with ventral striatopallidal parts of the basal ganglia in the rat.. J Comp Neurol.

[58] Kita H, Tachibana Y, Nambu A, Chiken S (2005). Balance of monosynaptic excitatory and disynaptic inhibitory responses of the globus pallidus induced after stimulation of the subthalamic nucleus in the monkey.. J Neurosci.

[59] Perea G, Araque A (2007). Astrocytes potentiate transmitter release at single hippocampal synapses.. Science.

[60] Goubard V, Fino E, Venance L (2011). Contribution of astrocytic glutamate and GABA uptake to corticostriatal information processing.. J Physiol.

[61] Villalba RM, Smith Y (2011). Neuroglial plasticity at striatal glutamatergic synapses in Parkinson's disease.. Front Syst Neurosci.

[62] Gradinaru V, Mogri M, Thompson KR, Henderson JM, Deisseroth K (2009). Optical deconstruction of parkinsonian neural circuitry.. Science.

[63] Beurrier C, Ben-Ari Y, Hammond C (2006). Preservation of the direct and indirect pathways in an in vitro preparation of the mouse basal ganglia.. Neuroscience.

[64] MacLean JN, Fenstermaker V, Watson BO, Yuste R (2006). A visual thalamocortical slice.. Nat Methods.

[65] Tymianski M, Bernstein GM, Abdel-Hamid KM, Sattler R, Velumian A (1997). A novel use for a carbodiimide compound for the fixation of fluorescent and non-fluorescent calcium indicators in situ following physiological experiments.. Cell Calcium.

[66] Preibisch S, Saalfeld S, Tomancak P (2009). Globally optimal stitching of tiled 3D microscopic image acquisitions.. Bioinformatics.

[67] Doube M, Klosowski MM, Arganda-Carreras I, Cordelieres FP, Dougherty RP (2010). BoneJ: Free and extensible bone image analysis in ImageJ.. Bone.

[68] Bolte S, Cordelieres FP (2006). A guided tour into subcellular colocalization analysis in light microscopy.. J Microsc.

[69] Platel JC, Dupuis A, Boisseau S, Villaz M, Albrieux M (2007). Synchrony of spontaneous calcium activity in mouse neocortex before synaptogenesis.. Eur J Neurosci.

[70] Mailly P, Charpier S, Mahon S, Menetrey A, Thierry AM (2001). Dendritic arborizations of the rat substantia nigra pars reticulata neurons: spatial organization and relation to the lamellar compartmentation of striato-nigral projections.. J Neurosci.

