# Supplementary figures and images for: Subthalamic Nucleus Electrical Stimulation Modulates Calcium Activity of Nigral Astrocytes
Source: PLoS One. 2012 Jul 27;7(7):e41793. doi: 10.1371/journal.pone.0041793 (PMC3407058; doi:10.1371/journal.pone.0041793)

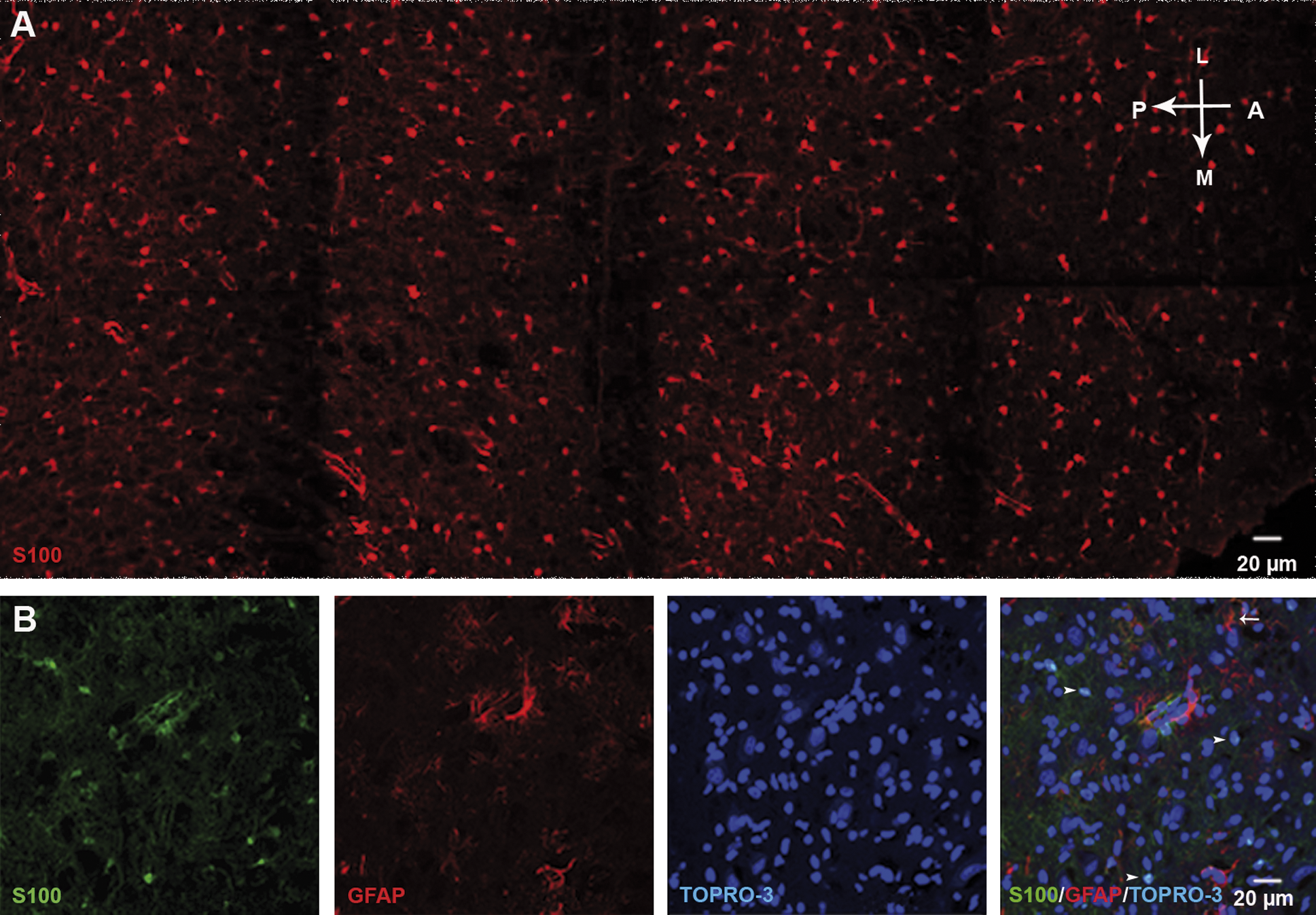

Supplement: Figure S1 — S100B and GFAP immunostainings in the SNr. (A) Two-photon multistack (9 slices at 2 µm spacing) mosaic reconstruction of S100B immunostaining in the SNr of a fixed brain slice. Antero-posterior (AP) and medio-lateral (ML) orientations are shown in the upper right part of the figure. (B) Confocal images of S100B (green) and GFAP (red) immunostained cells in the SNr of a sagittal section of rat brain. Merged image showing the proportion of S100B and GFAP positive cells (right panel). Nuclei are identified by TO-PRO staining (blue). Arrow indicates a GFAP positive cell that is S100B negative and arrowheads show several S100B-positive cells that are GFAP negative. (TIF) [file pone.0041793.s001.tif]
